# Specificity in the actions of the UBR1 ubiquitin ligase in the degradation of nuclear receptors^☆^

**DOI:** 10.1016/j.fob.2013.09.003

**Published:** 2013-09-17

**Authors:** Rasheda Sultana, Maria A. Theodoraki, Avrom J. Caplan

**Affiliations:** Department of Biology, The City College of New York, New York, NY 10031, USA

**Keywords:** Ubiquitin ligase, Quality control, Degradation, Molecular chaperone, Hsp90, UBR1, AR, androgen receptor, CHIP, C-terminal Hsp interacting protein, DMSO, dimethylsulfoxide, ER, estrogen receptor, GA, geldanamycin, GR, glucocorticoid receptor, HA, hemagglutinin, MEF, mouse embryonic fibroblasts, UPS, ubiquitin proteasome system

## Abstract

The UBR1 ubiquitin ligase promotes degradation of proteins via the N-end rule and by another mechanism that detects a misfolded conformation. Although UBR1 was shown recently to act on protein kinases whose misfolding was promoted by inhibition of Hsp90, it was unknown whether this ubiquitin ligase targeted other client types of the chaperone. We analyzed the role of UBR1 in the degradation of nuclear receptors that are classical clients of Hsp90. Our results showed that UBR1 deletion results in impaired degradation of the glucocorticoid receptor and the androgen receptor but not the estrogen receptor α. These findings demonstrate specificity in the actions of the UBR1 ubiquitin ligase in the degradation of Hsp90 clients in the presence of small molecule inhibitors that promote client misfolding.

## Highlights

• UBR1 promotes degradation of misfolded glucocorticoid receptors (GR) upon Hsp90 inhibition.
• Overexpression of UBR1 promotes degradation of the GR without Hsp90 inhibition.
• UBR1 also promotes degradation of the androgen receptor (AR) but not the estrogen receptor α (ERα).

## Graphical Abstract

**Figure:**
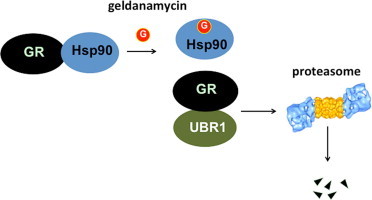

## Introduction

The intracellular environment does not favor protein stability. Newly made proteins fold under conditions that are relatively hot and crowed, which favor misfolding or aggregation rather than acquisition of the correct conformation [1]. These environmental conditions necessitated the evolution of a large cohort of genes that protect newly made proteins or promote degradation of those that misfold. The process that is governed by these genes is known variously as protein quality control or proteostasis [2,3]. The cellular machinery that comprises the proteostasis gene network includes molecular chaperones that interact with newly made or misfolded proteins [4] and components of the ubiquitin/proteasome system (UPS) for disposal of misfolded proteins [5]. In addition, the autophagic machinery facilitates disposal of insoluble aggregates that accumulate upon failure or imbalance of molecular chaperones or UPS components as cells age or upon stress [6]. The proteostasis network is sensitive to environmental conditions and adjusts the expression of a variety of genes to protect against proteotoxic stress [7].

The Hsp90 molecular chaperone sits at a hub of the proteostasis network by integrating folding of newly made proteins and degradation of misfolded ones. The balance of these triage decisions can be altered by small molecule inhibitors of Hsp90, which promote client protein degradation [8]. Many of these small molecules are in clinical trials as chemotherapeutic agents because of the role Hsp90 plays in the folding and degradation of oncogenic protein kinases and nuclear receptors [9].

Although it is clear that Hsp90 inhibition results in rapid degradation of client proteins via the UPS, the mechanisms underlying the clearance process are not well characterized. For example, the ubiquitin ligase C-terminal Hsp interacting protein (CHIP), plays a role in the degradation of several Hsp90 clients including protein kinases and nuclear receptors [10]. CHIP is not alone in its capacity to promote degradation of misfolded Hsp90 clients and several ubiquitin ligases have now been characterized to act in this pathway in yeast and mammalian cells [11]. What is unclear, however, is the specificity by which these ubiquitin ligases promote degradation when Hsp90 is inhibited. A good example of this is the glucocorticoid receptor (GR). GR can be ubiquitylated *in vitro* by CHIP [12] or upon CHIP overexpression [13], although degradation of this receptor is not affected by deletion of CHIP in cells [14], suggesting that other ubiquitin ligases act in a redundant manner. In this report, we show that the ubiquitin ligase UBR1 promotes degradation of the GR and the androgen receptor (AR) but not the estrogen receptor (ER) in cells treated with an Hsp90 inhibitor. This finding is significant in view of the direct role of the AR in promoting growth of prostate cancer cells.

## Materials and methods

### Materials

Geldanamycin was purchased from Invivogen (San Diego, CA), MG132 was purchased from Calbiochem (San Diego, CA), and both compounds were dissolved in 100% DMSO.

### Cell culture, transfection and plasmids

WT, Ubr1^−/−^ and Chip^−/−^ mouse embryonic fibroblast cells were maintained in DMEM medium supplemented with 10% heat-inactivated fetal Bovine serum (FBS) (Mediatech Inc., Herdon, VA), 100 units/ml penicillin, 100 μg/ml streptomycin (MP Biomedicals, LLC, France) and kept at 37 °C in 5% CO_2_ incubator. The transfection was performed as described [15]. For each transfection reaction 4 μg of plasmid DNA was used unless indicated. The plasmid encoding human AR, HA tagged GR and ER-α were gift from Dr. Michael J. Garabedian (New York School of Medicine). The plasmid encoding the rat UBR1 was a gift from Dr. Hiroshi Handa (Integrated Research Institute, Tokyo Institute of Technology, Yokohama, Japan). The WT, Ubr1^−/−^ cells were kind gift from Dr. Yong Tae Kwon (University of Pittsburg, Pennsylvania) and Chip^−/−^ cells from Dr. Cam Patterson (University of North Carolina).

### Western blotting and antibodies

Cells were transfected or grown to 70–80% confluence and exposed to DMSO, GA and MG132 for indicated time and dose. Lysates were prepared using lysis buffer containing 50 mM Tris pH 7.5, 2% SDS, .25% Na-deoxycholate, 150 mM NaCl, 1 mM EDTA, 10% glycerol, 1 mM phenylmethylsulfonyl fluoride, 10 mM NaF, 1 mM Na_3_VO_4_ and protease inhibitors (Complete mini, Roche Diagnostics, Indianapolis, IN). Lysates were sonicated for 3–4 times, 10 s each time. Protein concentration of lysates was determined using Bicinchoninic acid method (Pierce, Rockford, IL). Samples of 40 μg were analyzed in SDS–polyacrylamide gels and followed the same procedure described previously [15]. Antibodies used were: GR (MA1-510, Thermoscientific, Rockford, IL, USA), ER-α (sc-543, Santa Cruz Biotechnology, Inc.), AR [16]. PI3K (06-497, Millipore). Anti-HA (12CA5 form the Mount Sinai Hybridoma Facility, New York, NY) and UBR1 (Abcam Inc., Boston, MA).

## Results

In previous studies we demonstrated that mammalian UBR1 acted as part of the quality control apparatus that helps to clear misfolded protein kinases from cells treated with the Hsp90 inhibitor, geldanamycin (GA) [15]. UBR1 action was at least partially redundant with CHIP in this capacity. Based on these studies we tested whether UBR1 acted in the clearance of nuclear receptors, which represent another well-characterized class of Hsp90 client [17].

**Fig. 1:**
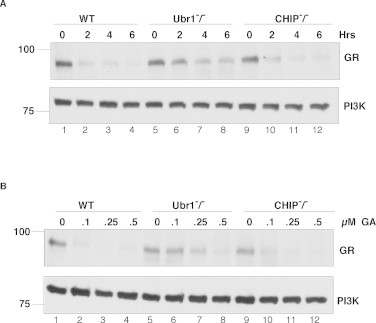
Geldanamycin dependent degradation of GR in WT, Ubr1^−/−^ and Chip^−/−^ MEF cells. (A) WT, Ubr1^−/−^ and CHIP^−/−^ MEF cells were treated with 100 nM of GA for indicated times. 40 μg of total protein from each cell line were analyzed by SDS–PAGE and probed with anti-GR; PI3K was used as a loading control. (B) WT, Ubr1^−/−^ and CHIP^−/−^ MEF cells were treated with different concentration of GA for 6 hours. 40μg of total protein from each cell line were fractionated by SDS–PAGE and probed with anti-GR. PI3K levels were analyzed as a loading control.

We first analyzed the ability of GA to promote degradation of the endogenously expressed GR in mouse embryonic fibroblasts (MEF). Treatment of cells with 100 nM GA resulted in a very rapid degradation of GR within a two-hour period and these levels were further diminished after 6 hours of treatment (Fig. 1A). GA also promoted degradation of GR in the UBR1^−/−^ cells but at a reduced rate, and there was still substantial GR remaining after 6 h of treatment. By contrast, CHIP^−/−^ cells behaved much like wild type MEFs with respect to GR degradation, as reported by others [14]. Similar findings were made in a dose–response assay where the levels of GR were barely affected at a dose of 100 nM in UBR1^−/−^ cells, whereas in wild type or CHIP^−/−^ MEFs the protein was mostly degraded (Fig. 1B). These findings suggest that UBR1 is important for the degradation of GR upon Hsp90 inhibition and that additional ubiquitin ligases distinct from CHIP also act in this capacity. We analyzed whether UBR2, a UBR1 homolog, acted in a similar capacity to UBR1 but found that GR degradation was similar in UBR2-/- cells and wild type MEFs (data not shown).

**Fig. 2:**
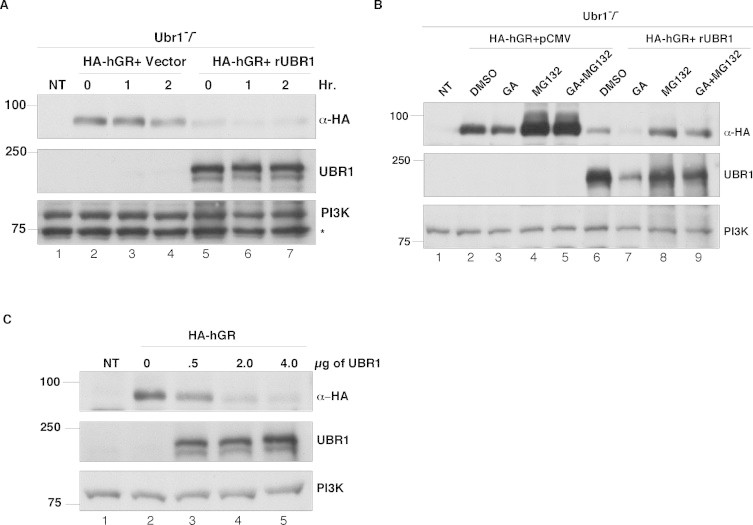
Effect of UBR1 overexpression on GR levels. (A) Ubr1^−/−^ cells were transfected with human HA-tagged GR (HA-hGR) and pCMV empty vector or rat UBR1. After 24 h of transfection the cells were treated with 50 nM of GA or DMSO for the indicated time points and harvested. 40 μg of cell lysates were analyzed in SDS–PAGE and probed with anti-HA (α-HA) and UBR1 antisera. PI3K was used as loading control. * in the PI3K blot indicates a non- specific band. (B) Ubr1^−/−^ cells were transfected with HA-hGR and empty vector or rat UBR1. After transfection cells were treated with DMSO or MG-132 (50 μM) for 18 h and GA (100 nM) for 6 h. Cells were harvested 24 h after transfection. 40 μg of cell lysates were analyzed in SDS–PAGE and probed with anti-HA (α-HA), UBR1 and PI3K. (C) Ubr1^−/−^ cells were transfected with HA-hGR and different amounts of rat E3 ligase UBR1 plasmid DNA (0, 0.5, 2 and 4 μg). Cell were harvested 24 h after transfection. 40 μg of total cell lysates were fractionated by SDS–PAGE and probed with anti-HA (α-HA) and UBR1 antisera. PI3K was used as a loading control. NT represents non-transfected Ubr1^−/−^ MEF cells in A–C.

To determine the UBR1 dependence on the degradation phenotype of UBR1^−/−^ cells, we used transfection to introduce wild type rat UBR1 (rUBR1) into the cells. An HA-tagged version of GR (HA-GR), or an empty vector as a control were co-transfected into UBR1^−/−^ cells (Fig. 2A). The effect of rUBR1 overexpression was to cause a large reduction in the levels of the transfected HA-GR even without GA treatment. Furthermore, treatment with GA did not lead to a substantial decrease in the HA-GR levels after 2 h. These findings suggested that HA-GR was sensitive to the levels of rUBR1 even in the absence of GA. The effect of GA was probed by a more prolonged treatment of 18 h (Fig. 2B), and in this case we observed that the GA-sensitivity of the transfected HA-GR was restored. Note also that rUBR1 levels were also sensitive to GA treatment as described previously [15]. HA-UBR1 levels were restored upon treatment with the proteasome inhibitor, MG132, indicating that UBR1 was acting via the UPS as expected, although this also occurred in the vector-transfected control cells, thus confirming redundancy in UBR1 action.

The large decrease in HA-GR levels upon co-transfection of rUBR1 (Fig. 2A) was studied further in a dose–response experiment (Fig. 2C). In this case we observed a direct correlation between increasing rUBR1 concentration and decreasing HA-GR levels, further suggesting that UBR1 controls GR levels both in the absence and presence of functional Hsp90.

**Fig. 3:**
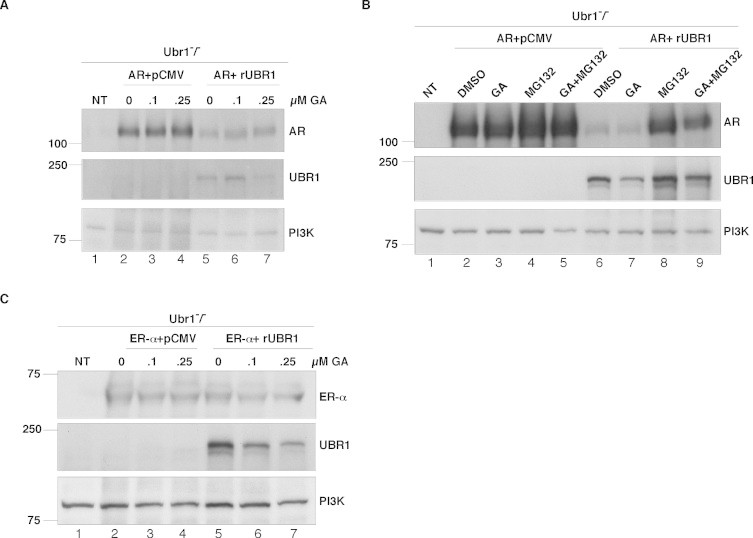
Effects of UBR1 overexpression on AR and ER-α. (A) Ubr1^−/−^ cells were transfected with the AR expressing plasmid and pCMV empty vector or rat UBR1. After 24 h of transfection the cells were treated with different concentrations of GA or vehicle for 24 h. 40 μg of cell lysates were analyzed in SDS–PAGE and probed with AR and UBR1 antisera. PI3K was used as loading control. (B) Ubr1^−/−^ cells were transfected with AR and pCMV empty vector or rat UBR1. After transfection the cells were treated with DMSO, MG-132 (50 μM) for 18 h and GA (100 nM) for 6 h. The cells were harvested 24 h after transfection and 40 μg of cell lysates were analyzed by SDS–PAGE and probed with anti AR, UBR1 and PI3K. (C) Ubr1^−/−^ cells were transfected a plasmid expressing ER-α or pCMV empty vector or rat UBR1. After 24 h of transfection, the cells were treated with different concentrations of GA or DMSO for 24 h. 40 μg of cell lysates were analyzed by SDS–PAGE and probed with AR and UBR1 antisera. PI3K was used as loading control. NT represents non-transfected Ubr1^−/−^ MEF cells in A–C.

The results described thus far suggest that UBR1 promotes degradation of GR and further studies addressed the specificity of that function with analysis of the human androgen receptor (hAR) and estrogen receptor α (hERα). hAR was transfected into the UBR1^−/−^ cells with either the plasmid overexpressing rat UBR1 or an empty vector. The findings (Fig. 3A) were very similar those observed for transfection of HA-GR (Fig. 2). In this case, there was a substantial decrease in hAR levels when rUBR1 was co-overexpressed in the UBR1^−/−^ cells compared with vector alone even in the absence of GA. In both cases, however, there was little further effects on degradation of hAR by GA treatment (Fig. 3A and B), although rUBR1 levels were sensitive at the highest treatment level of GA. Treatment of these cells with MG132, however, led to a strong resurgence of hAR levels that was particularly evident in the cells co-expressing rUBR1. These data strongly support a role for UBR1 in the quality control of hAR levels. By contrast, similar experiments with transfected human hERα suggest that UBR1 does not play a similar role in the degradation of this nuclear receptor (Fig. 3C). Co-transfection of rUBR1 did not affect hERα in the same manner it did with GR and hAR. Our combined findings therefore suggest specificity in the actions of UBR1 in the clearance of GR, AR and ER, with the AR displaying the most dependence on UBR1 actions.

## Discussion

Quality control ubiquitin ligases promote degradation of misfolded proteins. In yeast there exists a small network of quality control ubiquitin ligases that function in the degradation of Hsp90 clients that includes UBR1 and at least 7 others acting in the cytosol and the nucleus [11]. In mammalian cells, there are three ubiquitin ligases that are known to facilitate the clearance of protein kinases that misfold upon Hsp90 inhibition and these include CHIP, CUL5 and UBR1 (reviewed in [8]). It is striking, however, that neither CHIP nor CUL5 affect the degradation of GR when they are deleted or down-regulated [14,18], supporting the hypothesis that redundant ubiquitin ligases exist. In the studies presented in this report we identify UBR1 as having this specific function in GR degradation, and also show that UBR1 acts in the clearance of the hAR, but not hERα.

UBR1 is a well-characterized ubiquitin ligase that functions via N-end rule degradation and also via non-N-end rule pathways in protein quality control [19]. In yeast, Ubr1 acts via non-N-end rule pathways to promote degradation of misfolded proteins [20–22] and it seems likely that this will be the case for mammalian UBR1 based on its function in the clearance of nuclear receptors, as shown here, and protein kinases [15] that misfold upon Hsp90 inhibition. Furthermore, dipeptides [23,24] known to inhibit degradation via the N-end rule (Leu-Ala and Arg-Ala) had no effect on the degradation of GR and AR (data not shown).

As mentioned above, yeast E3s that act in cytosolic quality control are highly redundant. In mammalian cells there is also some redundancy in the actions of E3 ligases in cellular quality control. In a previous study we observed redundancy between CHIP and UBR1 for the degradation of CDK4 and AKT protein kinases [15]. By contrast, there does not appear to be a role for CHIP in the degradation of GR that misfolds due to Hsp90 inhibition. CHIP can promote GR degradation when it is overexpressed, but as discovered by others, CHIP knockout cells have no impairment in misfolded GR degradation [14]. In this case, therefore, there appears to be specificity in E3 action. This specificity is further supported by our finding that overexpression of UBR1 had no effect on the degradation of ER-α, whereas downregulation of CHIP does inhibit ER-α degradation [25].

In conclusion, our findings provide further support for a conserved role for UBR1 in the degradation of misfolded proteins in mammalian cells. The finding that UBR1 acts on some misfolded nuclear receptors suggests it may have a role to play in a variety of human diseases of aging including cancer as well as Johanson–Blizzard syndrome [26].
